# Knowledge, attitudes, and practices regarding proctological diseases with a focus on anal fistula: A cross-sectional study from a tertiary care center in Turkiye

**DOI:** 10.1097/MD.0000000000049258

**Published:** 2026-07-17

**Authors:** Can Sahin, Hasan Bostanci

**Affiliations:** a Department of General Surgery, Yenimahalle Training and Research Hospital, Ankara, Turkiye; b Department of General Surgery, Gazi University Faculty of Medicine, Ankara, Turkiye.

**Keywords:** attitudes, fistula-in-ano, knowledge, practices, proctology

## Abstract

Globally, sociocultural factors often pose significant barriers for patients in seeking medical attention and accessing appropriate treatment. This study aims to evaluate the knowledge, attitudes, and practices regarding anal fistula and other proctological conditions in Turkiye. The findings will help identify gaps in health education and support the development of targeted public health interventions. This research is a cross-sectional survey study conducted among individuals who applied to a tertiary care center. Participants were consecutively recruited and consisted of individuals aged 18 and over who visited the general surgery outpatient clinic for any reason. Data were collected through an online questionnaire based on Google Forms. The survey included questions on demographic information, disease awareness, attitudes toward treatment, and healthcare-seeking behavior. A knowledge, attitudes, and practices (KAP) analysis was performed to assess participants’ understanding of the disease, their treatment preferences, and health-related practices. A total of 702 participants were included in the analysis, of whom 377 (53.7%) were female and 325 (46.3%) were male. A history of at least one proctological disease was reported by 332 individuals (47.2%), and 115 participants (16.3%) had undergone surgery for a proctological condition at least once. Traditional treatment methods had been tried by 73 respondents (10.3%). A total of 330 participants (46.9%) stated they were aware of the causes of anal fistula; however, only 143 (43.3%) of them gave correct answers. The total KAP score was significantly lower among participants with primary education compared with those with higher education (*P* = .02). Participants over the age of 45 had significantly higher total KAP scores compared with both younger age groups (≤30 and 31–45 years; *P* < .001). In addition, participants with a history of proctological disease or a diagnosis of anal fistula had significantly higher KAP scores (*P* < .05). Raising awareness of proctological diseases in Turkiye remains a significant challenge. On the basis of the findings of the KAP analysis, it is essential to identify knowledge gaps and misconceptions and to develop educational and public health strategies aimed at improving community health outcomes.

## 1. Introduction

The sociological structure and level of development of a country directly influence the population’s ability and determination to access appropriate and modern medical treatment. This effect becomes particularly evident in conditions such as proctological diseases, where patients may feel hesitation or reluctance in seeking medical care. The management of proctological diseases is often challenging, especially in complex cases, because of high recurrence rates. Recurrence is more likely when treatment is performed by inexperienced practitioners. Achieving satisfactory outcomes in these patients requires timely access to appropriate and effective treatment. In many cases, surgical intervention remains the only definitive therapeutic option.

Proctological diseases, such as hemorrhoidal disease, anal fissure, perianal abscess, and anal fistula, are commonly encountered in general surgery practice and may lead to pain, bleeding, discharge, impaired quality of life, repeated healthcare visits, and loss of productivity. Although population-based data on their incidence and prevalence in Turkiye are limited, these conditions represent an important clinical burden. In addition, embarrassment, social stigma, fear of surgery, and reliance on traditional remedies may delay timely diagnosis and appropriate treatment. Therefore, assessing public knowledge, attitudes, and practices (KAP) is important for identifying misconceptions and guiding targeted awareness programs.

The treatment of hemorrhoids dates back to ancient times, with the earliest recorded methods appearing in Egyptian and Greek medical texts. Over the centuries, various techniques – including ligation, cauterization, and surgical excision – have evolved, reflecting the ongoing search for effective and durable solutions.^[1]^ The treatment of anal fistula has evolved over time, progressing toward sphincter-preserving techniques in modern clinical practice.^[2]^ Traditional remedies, particularly for conditions like hemorrhoids, continue to be widely used among the general population despite limited clinical efficacy.^[3]^ Traditional treatment approaches have been used for nearly all types of proctological diseases in countries such as China, India, and Iran, with many of these methods still in use among the general population today. Similarly, in Turkiye, practices such as wet cupping (hijama) and various herbal therapies are commonly used by the public, often without adequate medical guidance.^[4–7]^

KAP analyses offer a systematic way to assess public knowledge, beliefs, and behavioral patterns regarding health. Especially in conditions where traditional treatments are commonly used, these analyses help identify knowledge gaps and entrenched attitudes, contributing to the development of targeted education and intervention programs. KAP studies are considered practical and effective tools in public health planning. Their structured approach allows researchers to generate actionable insights that can guide culturally sensitive healthcare strategies.^[8]^

Although certain traditional methods have found limited space in the literature for the management of selected proctological conditions, there is no evidence supporting their efficacy in diseases like anal fistula, where surgical intervention is often essential.^[9]^ Nevertheless, in developing and underdeveloped countries, these easily accessible treatments are frequently preferred by the public. This trend may contribute to disease progression, delay in seeking appropriate treatment, and increased therapeutic complexity, ultimately posing a serious public health concern.

Despite the increasing clinical burden of proctological diseases, there is a limited understanding of KAP in the population regarding these conditions, particularly anal fistula. Existing studies have primarily focused on hemorrhoidal disease and have not comprehensively evaluated awareness levels, treatment preferences, and healthcare-seeking behaviors across different demographic groups. Therefore, a clear knowledge gap remains regarding how individuals perceive anal fistula and related proctological conditions, especially in terms of misconceptions and reliance on traditional practices.^[10,11]^

This study aimed to assess the KAP of adult participants regarding proctological diseases, with a particular focus on anal fistula, in a tertiary care center in Turkiye. The specific objectives were to evaluate participants’ knowledge about the causes and symptoms of anal fistula, to assess their attitudes toward modern medical/surgical treatment and traditional remedies, to determine their healthcare-seeking practices, and to analyze the association between total KAP scores and sociodemographic or clinical factors, including age, sex, education level, and history of proctological disease.

## 2. Materials and methods

### 2.1. Study design

This study was designed as a descriptive, cross-sectional survey aimed at evaluating the KAP regarding proctological conditions. The research was conducted over a 6-month period between December 2024 and May 2025 at a tertiary care center in Turkiye.

### 2.2. Setting and participants

The study population consisted of adult individuals aged 18 and over who visited the general surgery outpatient clinic for any reason. A total of 754 individuals participated in the survey. However, 52 responses were excluded because of incomplete data or because the respondent was under 18 years of age. As a result, 702 participants were included in the final analysis.

Inclusion criteria were being 18 years of age or older, visiting the general surgery outpatient clinic for any reason during the study period, being able to read and understand the Turkish questionnaire, and voluntarily agreeing to participate in the study. Exclusion criteria were being younger than 18 years of age, submitting incomplete questionnaire responses, and being unable to read or understand the questionnaire sufficiently to provide reliable answers.

The minimum sample size was estimated using a standard formula for cross-sectional studies. In the absence of prior data, a conservative proportion of 50% was assumed. With a 95% confidence level and 5% margin of error, the required sample size was 384. As 702 participants were included, the sample size was sufficient.

### 2.3. Data collection

Data were collected using a structured, self-administered questionnaire prepared in Google Forms format. The survey included sections addressing sociodemographic characteristics, awareness of disease etiology, attitudes toward treatment options, and healthcare-seeking behavior. A KAP analysis was performed to assess participants’ understanding of proctological diseases, their treatment preferences, and real-life practices.

### 2.4. KAP scoring system

To assess participants’ understanding and behavior regarding proctological conditions, a structured KAP questionnaire was developed based on previously validated tools in public health research and adapted from existing studies because of the lack of a comprehensive instrument covering all proctological diseases.^[10–12]^ The questionnaire was structured into 3 domains: knowledge, attitudes, and practices. The knowledge section included items assessing awareness of disease etiology and symptoms of anal fistula. The attitudes domain evaluated participants’ perceptions of treatment options, including preference for modern medical care versus traditional methods, primarily in the context of anal fistula. In contrast, the practices domain was designed to encompass a broader spectrum of proctological diseases and assessed real-life health-seeking behaviors, such as consulting a physician, receiving treatment, and the use or avoidance of nonmedical remedies. The questionnaire included multiple-choice and Likert-scale items covering these domains (knowledge: 2 items; attitudes: 5 items; practices: 4 items). Knowledge was scored by assigning 1 point per correct response regarding causes and symptoms of anal fistula. Attitudes were assessed on a 4-point scale, with higher scores indicating greater acceptance of modern medical treatment and less preference for traditional methods. Practice was scored based on reported history of disease and actions taken, with 1 point assigned for visiting a doctor, receiving medication, undergoing surgery, and refusing unverified traditional treatments. The total KAP score ranged from 5 to 26, with higher scores reflecting more accurate knowledge, positive health-seeking behavior, and evidence-based attitudes.

### 2.5. Ethical approval

All procedures performed in this study were in accordance with the ethical standards of the institutional and/or national research committee and the 1964 Helsinki Declaration and its later amendments or comparable ethical standards. This study was approved by The Local Ethical Committee of Gazi University (reference: 26.11.2024-1829). Informed consent was obtained from all individual participants included in the study.

### 2.6. Statistical analysis

Statistical analyses were performed using IBM SPSS Statistics version 23.0. Descriptive statistics were reported as means and standard deviations for continuous variables, and frequencies and percentages for categorical variables. The normality of distribution for continuous variables was assessed using the Shapiro–Wilk test. For comparisons between 2 independent groups, the independent samples *t* test was used when parametric assumptions were met, and the Mann–Whitney *U* test was applied otherwise. For comparisons involving more than 2 groups, one-way analysis of variance or the Kruskal–Wallis test was used depending on data distribution. Levene test was performed to evaluate homogeneity of variances. Statistical significance was defined as a *P* < .05.

## 3. Results

Of 702 patients, 377 (53.7%) were female and 325 (46.3%) were male, with a mean age of 38.52 years (range: 18–73). Regarding educational background, 14 participants (2.0%) had completed only primary school, 20 (2.8%) had completed middle school, 164 (23.4%) had a high school education, and 504 participants (71.8%) held a higher education degree (Table 1).

**Table 1:** Demographic characteristics of the study population.

Characteristics	n	%
Age (yr)	38.52 ± 13.24 (18–73)	
Gender, n (%)		
** **Male	325	46.3
** **Female	377	53.7
Education		
** **Primary school	14	2
** **Middle school	20	2.8
** **High school	164	23.4
** **Higher school	504	71.8

A history of at least one proctological disease was reported by 332 individuals (47.2%). Among these participants, hemorrhoidal disease was the most frequently reported condition (n = 158, 47.6%), followed by anal fissure (n = 99, 29.8%), anal fistula (n = 65, 19.6%), pilonidal sinus (n = 50, 15.1%), anal abscess (n = 29 8.7%), and anal condyloma (n = 3, 0.9%; Table 2).

**Table 2:** Distribution of reported proctological diseases among affected participants (n = 332).

Proctological disease (n = 332)	n	%
Hemorrhoidal disease	158	47.6
Anal fissure	99	29.8
Anal fistula	65	19.6
Pilonidal sinus	50	15.1
Anal abscess	29	8.7
Anal condyloma	3	0.9

Among the 332 participants who reported having experienced at least one proctological disease, 80 individuals (24.0%) stated that they had tried nonmedical treatment methods. These approaches were often based on traditional or folk remedies, including the application of substances such as diesel oil, pan-fried egg, eggplant stem, cooked onion, and Turkish delight to the anal region, and wet cupping (hajamat) was also commonly reported. In addition, 330 participants (46.9%) reported that they were aware of the causes of anal fistula; however, only 143 (43.3% of those who reported awareness) provided correct responses. This finding indicates a discrepancy between perceived and actual knowledge among participants.

Participants’ total scores ranged from 5 to 26 points, derived from their responses to questions categorized into 3 main domains: knowledge, attitudes, and practices. Knowledge items evaluated participants’ recognition of anal fistula causes and symptoms, attitude items explored their acceptance of modern versus traditional treatment approaches, and practice items assessed healthcare-seeking behaviors and avoidance of nonmedical interventions. The distribution of responses, including counts and percentages for each question, is presented in Table 3.

**Table 3:** Distribution of KAP scores among participants.

Knowledge, n (%)	Yes (1 points)		No (0 points)	
Identify anal abscess as a cause of anal fistula	147 (20.9%)		555 (79.1%)	
Identify pain, discharge, or swelling as symptoms of anal fistula	368 (52.4%)		334 (47.6%)	
Attitudes, n (%)	Strongly agree (4 points)	Agree (3 points)	Disagree (2 points)	Strongly disagree (1 points)
Would accept modern medical/surgical options if diagnosed with anal fistula	354 (52%)	294 (43.2%)	22 (3.2%)	11 (1.6%)
Would recommend modern medical/surgical options to relatives	347 (51.3%)	307 (45.4%)	15 (2.2%)	7 (1.0%)
Would not try traditional treatments if personally affected	96 (14.1%)	243 (35.7%)	268 (39.4%)	73 (10.7%)
Would not recommend traditional treatments to relatives	88 (12.9%)	280 (41.1%)	252 (37.0%)	62 (9.1%)
Believe surgery is superior to traditional methods	153 (22.5%)	383 (56.35%)	110 (16.2%)	34 (5.0%)
Practices, n (%)	Yes (1 points)		No (0 points)	
Visited a doctor for a proctological disease	274 (39%)		428 (61%)	
Received medical treatment for a proctological disease	245 (34.9%)		457 (65.1%)	
Undergone surgery for a proctological disease	115 (16.4%)		587 (83.6%)	
Did not try traditional methods	622 (88.6%)		80 (11.4%)	

KAP = knowledge, attitudes, and practices.

**Figure 1.**
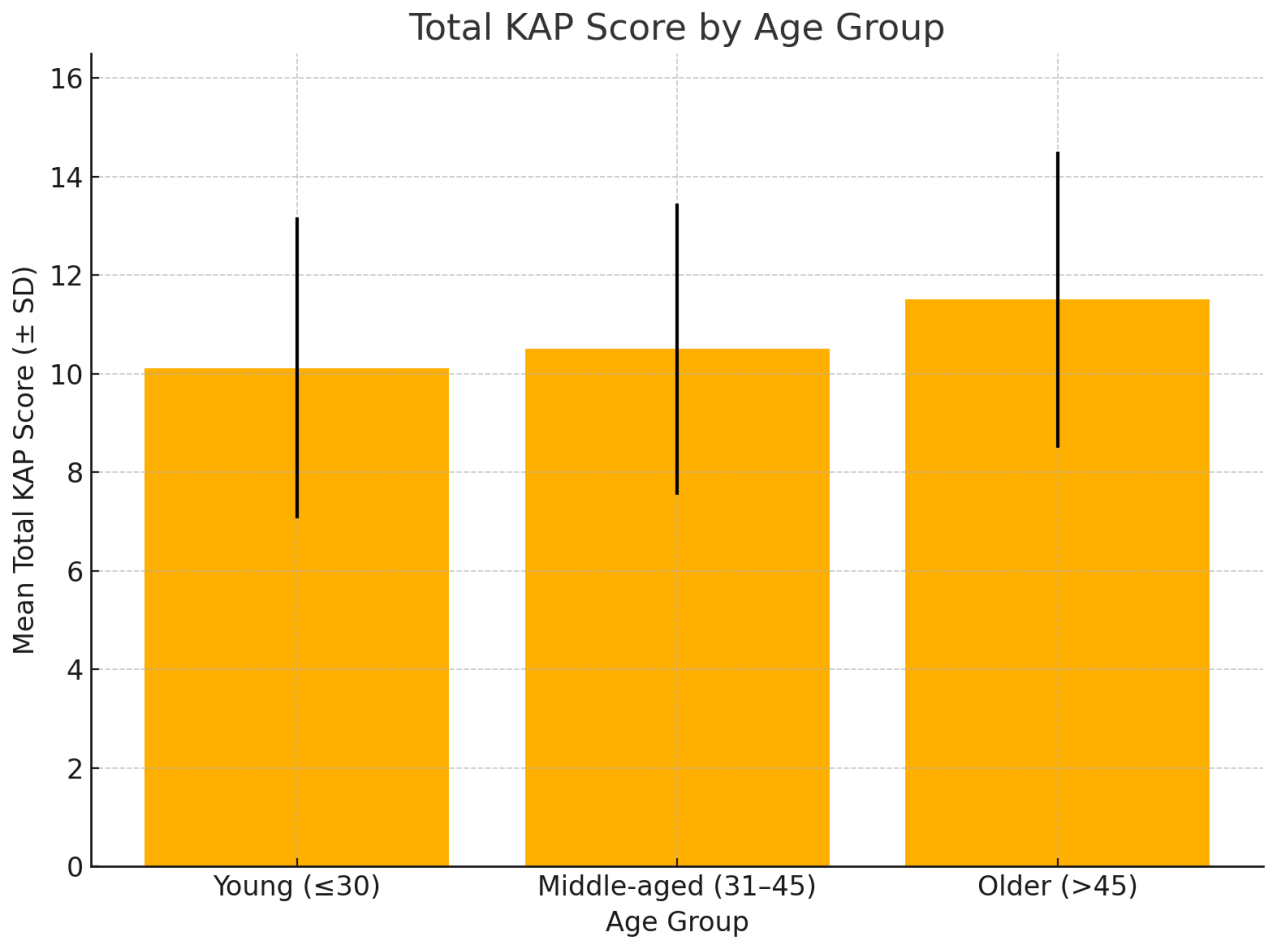
Comparison of mean total KAP scores across age groups (≤30, 31–45, >45 years). KAP = knowledge, attitudes, and practices, SD = standard deviation.

**Figure 2.**
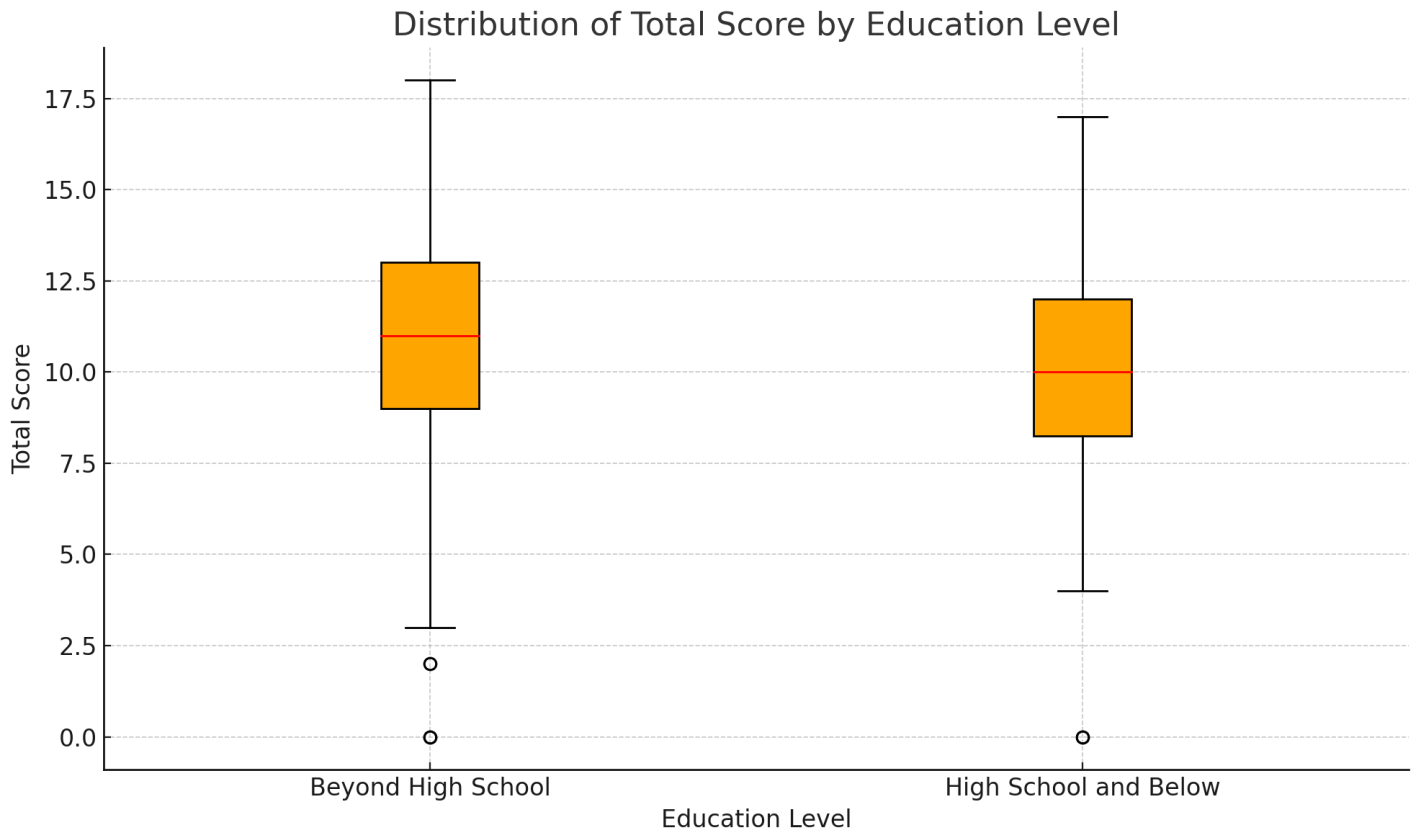
Distribution of total KAP scores according to educational level. KAP = knowledge, attitudes, and practices.

The overall mean KAP score was 10.7 ± 3.1, indicating a moderate level of KAP regarding proctological diseases among the study population. The mean total KAP score differed significantly across age groups, with participants aged >45 years scoring higher compared to those aged ≤30 years and 31 to 45 years (*P* < .001; Fig. 1). Gender did not show a statistically significant association with KAP scores (*P* = .08). Participants with education beyond high school had higher mean scores compared to those with high school education or below (*P* = .02; Fig. 2). A history of any proctological disease was associated with a markedly higher mean score compared to those without such a history (*P* < .001). Similarly, participants with a history of perianal fistula had significantly higher scores compared to those without (*P* < .001), and when compared specifically to patients with other proctological diseases (*P* = .01; Table 4).

**Table 4:** KAP score variations across sociodemographic and clinical factors.

Variables	Number		Percentage (%)	KAP score (mean ± SD)	*P* value
Age					
** **≤30 yr	252		35.9	10.12 ± 3.05	**<.001**
** **31–45 yr	226		32.19	10.50 ± 2.95	
** **>45 yr	224		31.91	11.50 ± 3.01	
Gender					
** **Male	325		46.3	10.89 ± 3.25	.08
** **Female	377		53.7	10.50 ± 2.88	
Education					
** **High school and below	198		28.21	10.09 ± 2.88	**.02**
** **Beyond high school	504		71.79	10.86 ± 3.07	
Any proctological disease history					
** **Yes	332		47.29	12.29 ± 2.86	**<.001**
** **No	370		52.71	9.24 ± 2.46	
Perianal fistula history					
** **Yes	65		9.26	13.09 ± 2.5	**<.001**
** **No	637		90.74	10.44 ± 3.01	
Perianal fistula vs other proctological diseases					
** **Fistula	65		19.58	13.09 ± 2.5	**.01**
** **Others	267		80.42	10.95 ± 3.01	
Overall	702	100		10.7 ± 3.1	**–**

Bold values indicate statistically significant results (*P* < .05).

KAP = knowledge, attitudes, and practices, SD = standard deviation.

A total of 470 participants (66.9%) expressed interest in obtaining more detailed information about anal fistula. Among these, the majority preferred to receive information directly from healthcare professionals (72.8%), followed by social media or internet sources (22.6%), brochures (7.9%), and television/radio media (3.8%).

## 4. Discussion

Proctological diseases are often neglected conditions within communities, frequently associated with embarrassment and stigma. Consequently, patients may avoid professional medical care and instead rely on misconceptions or unproven traditional remedies, reflecting the poor awareness of the population and creating barriers to timely and effective treatment. KAP analyses aimed at assessing public awareness have rarely been applied in the field of proctology, with only a few studies focusing on hemorrhoidal disease.^[10,11]^ Anal fistula, in particular, remains one of the least recognized conditions and is highly susceptible to being managed with inappropriate or misleading traditional methods. However, to the best of our knowledge, no previous study has specifically examined this disease from such a perspective.

Previous studies applying the KAP framework to proctological conditions have been largely limited to hemorrhoidal disease. For example, Ouattara et al^[10]^ reported that patients in Côte d’Ivoire demonstrated poor knowledge and widespread misconceptions about hemorrhoids, which contributed to delays in seeking appropriate medical care. Similarly, Abed et al^[11]^ highlighted that inadequate awareness and suboptimal attitudes following hemorrhoid surgery negatively influenced patients’ quality of life and treatment satisfaction. Unlike these studies, which did not evaluate demographic influences on KAP scores, our research provides a more detailed analysis by comparing age, gender, and educational background. We demonstrated that younger participants and those with lower levels of education had significantly poorer awareness, whereas patients with a history of proctological disease – particularly anal fistula – achieved higher scores. This represents a novel contribution, as it not only highlights the limited awareness of anal fistula in the general population but also identifies the subgroups most in need of targeted educational interventions.

A study from Jordan demonstrated that cultural beliefs and superstitions negatively influence treatment adherence in chronic diseases, with higher education improving adherence and lower education, female gender, and older age associated with stronger superstitious thinking. Similar patterns were observed in our study, where limited awareness and reliance on traditional practices in proctological diseases were associated with inappropriate treatment choices and delays in seeking proper care.^[13]^

Ndzengue et al^[14]^ reported that anal diseases are often perceived as taboo and neglected within society, leading to delays in diagnosis and treatment. In addition to common conditions such as hemorrhoids and fissures, suppurative diseases including anal fistulas and abscesses were also described as constituting a significant burden. These findings are consistent with the results of our study, in which we observed low awareness of proctological diseases in the community and a tendency to rely on anecdotal or traditional remedies rather than modern medical solutions.

Simões et al^[15]^ conducted a study in Brazil which revealed that public awareness regarding proctology was extremely low. In their survey, 86% of participants stated that they did not know what proctology was, and only 10.5% were able to provide the correct definition. Moreover, most respondents with incorrect knowledge associated proctology with the prostate. The authors emphasized that this lack of knowledge may be attributed to social taboos, prejudice, and insufficient educational efforts from healthcare professionals. In our study, we similarly identified poor public awareness of proctological diseases, and the survey results demonstrated that the majority of participants expressed a preference to be directly informed by healthcare professionals.

In our country, as in many others, the frequent use of traditional remedies persists even in conditions like anal fistula, where surgical intervention is almost universally required. It is particularly striking that some participants in our survey reported applying substances such as fried egg, diesel oil, sugar, and cooked eggplant to the anal region – practices that reflect deeply rooted cultural beliefs rather than evidence-based approaches. Traditional treatments are often more accessible and lower in cost compared with modern medical care, which partially explains their popularity, especially among individuals from low-income or rural backgrounds. In addition, traditional methods typically do not require hospitalization, minimizing disruption to patients’ social and economic lives.^[16]^ Another contributing factor may be the fear of complications associated with surgical management, such as sphincter damage, recurrence, and incontinence, which leads some patients to avoid formal medical care altogether.^[17]^

Interestingly, our analysis revealed a statistically significant increase in KAP scores with advancing age – an unexpected finding, as we had initially hypothesized lower awareness in older individuals. We believe this trend may be explained by increased personal or secondhand experience with proctological conditions over time, leading to greater awareness and informed decision-making. Similarly, as expected, higher levels of education were associated with improved KAP scores. Notably, participants with a prior history of anal fistula or other proctological conditions also demonstrated significantly higher scores, which we attribute to the educational impact of undergoing successful modern treatment. To our knowledge, this is the first study in the literature to assess KAP scores in relation to demographic variables within the context of proctological diseases. By addressing a previously unexplored area, our findings provide valuable insights that may inform future public health education and intervention strategies.

On the basis of these findings, several concrete steps may be considered to improve public awareness and health system responsiveness regarding proctological diseases. First, culturally adapted patient education materials should be developed to address common misconceptions, stigma, and inappropriate traditional practices. Second, primary care physicians should be supported through targeted training programs to improve counseling on proctological symptoms and timely referral pathways. Third, public health campaigns should particularly target younger individuals and those with lower educational levels, as these groups demonstrated lower KAP scores in the present study. Finally, proctological awareness may be integrated into broader national health literacy programs to promote early healthcare-seeking behavior and reduce delays in appropriate treatment.

This study has certain limitations. First, the study population consisted of individuals attending a hospital outpatient clinic, which may not be representative of the general population and may have led to an overestimation of KAP scores. Second, the data were collected through self-reported questionnaires; therefore, the findings may be affected by recall bias, socially desirable responses, and may not fully reflect actual health-related behavior. In addition, the questionnaire used in this study, although developed based on previously published studies, was not formally validated or pilot-tested before implementation, which may affect the generalizability of the findings. The limited number of knowledge items may not fully reflect participants’ overall knowledge of proctological diseases, and the composite KAP score may have been influenced by disproportionate weighting across the knowledge, attitude, and practice domains. Since multivariable analysis was not performed, potential confounding factors could not be fully controlled. Cultural and religious variables, which may influence treatment preferences and traditional practices, were also not specifically assessed. Although the questionnaire was not generally administered to physicians or nurses, healthcare workers could not be completely excluded from the study population, which may have influenced the KAP scores. Finally, while this study provides valuable preliminary insights, multicenter studies with diverse populations are needed to further validate and enrich these findings.

## 5. Conclusion

KAP studies in the field of proctology remain limited, and to the best of our knowledge, no study has specifically focused on anal fistula. This study therefore represents a novel contribution, providing the first assessment of KAP in this area. By examining a large hospital-based sample, our findings reveal important gaps in awareness, misconceptions regarding traditional practices, and variations in health-seeking behaviors across demographic groups. These results underline the need for targeted educational strategies and effective public health interventions to correct misconceptions and improve timely access to appropriate care.

## Author contributions

**Conceptualization:** Can Sahin.

**Data curation:** Can Sahin.

**Investigation:** Can Sahin.

**Resources:** Can Sahin.

**Methodology:** Hasan Bostanci.

**Supervision:** Hasan Bostanci.

**Writing – original draft:** Can Sahin.

**Writing – review & editing:** Hasan Bostanci.
